# Genome-wide DNA methylation profiles regulate distinct heat stress response in zebu (*Bos indicus*) and crossbred (*Bos indicus* × *Bos taurus*) cattle

**DOI:** 10.1016/j.cstres.2024.06.005

**Published:** 2024-06-25

**Authors:** Basavaraj Sajjanar, Mohd Tanzeel Aalam, Owais Khan, Sujoy K Dhara, Jyotirmoy Ghosh, Ravi Kumar Gandham, Praveen K Gupta, Pallab Chaudhuri, Triveni Dutt, Gyanendra Singh, Bishnu Prasad Mishra

**Affiliations:** 1 ICAR-Indian Veterinary Research Institute, Izatnagar, Bareilly, Uttar Pradesh, India; 2 ICAR-Indian Veterinary Research Institute, Bengaluru Campus, Bengaluru, Karnataka, India; 3 ICAR-National Institute of Animal Nutrition and Physiology, Bengaluru, Karnataka, India; 4 ICAR-National Bureau of Animal Genetic Resources, Karnal, Haryana, India

**Keywords:** Temperature, DNA methylation, WGBS, Gene expression, Stress responses

## Abstract

Epigenetic variations result from long-term adaptation to environmental factors. The *Bos indicus* (zebu) adapted to tropical conditions, whereas *Bos taurus* adapted to temperate conditions; hence native zebu cattle and its crossbred (*B indicus* × *B taurus*) show differences in responses to heat stress. The present study evaluated genome-wide DNA methylation profiles of these two breeds of cattle that may explain distinct heat stress responses. Physiological responses to heat stress and estimated values of Iberia heat tolerance coefficient and Benezra's coefficient of adaptability revealed better relative thermotolerance of Hariana compared to the Vrindavani cattle. Genome-wide DNA methylation patterns were different for Hariana and Vrindavani cattle. The comparison between breeds indicated the presence of 4599 significant differentially methylated CpGs with 756 hypermethylated and 3845 hypomethylated in Hariana compared to the Vrindavani cattle. Further, we found 79 genes that showed both differential methylation and differential expression that are involved in cellular stress response functions. Differential methylations in the microRNA coding sequences also revealed their functions in heat stress responses. Taken together, epigenetic differences represent the potential regulation of long-term adaptation of Hariana (*B indicus*) cattle to the tropical environment and relative thermotolerance.

## Introduction

Epigenetic changes are one of the major mechanisms to regulate gene expression patterns and, in turn, have implications for a wide range of cellular functions. DNA methylation, histone modifications (acetylation, phosphorylation, methylation, and ubiquitination), and noncoding ribosomal RNA (RNA) constitute epigenetic regulations.^1^ Environmental factors are known to shape the epigenetic changes. Hence, epigenetic modifications potentially connect the outside environment to the genome for gene expression and phenotypic response.^2^ Long-term adaptation to certain environmental conditions may be associated with specific epigenetic variations like DNA methylations in the genome.^3^ For example, adaptation to hydrogen sulfide-rich springs was associated with distinct DNA methylation patterns in the *Poecilia mexicana* fish.^4^ Similarly, differential DNA methylations were observed in small ruminants (sheep and goats) that were reared in two distinct climatic conditions.^5^

Zebu cattle (*Bos indicus*) are mainly originated in the Indian subcontinent and farmed throughout tropical countries. Taurine cattle (*Bos taurus*) are found primarily in Europe and other temperate areas of the Americas and Australia. Earlier, these two populations have been compared for multiple traits.^6^ The phenotypic differences between zebu and taurine cattle are broadly related to the metabolic and endocrine features that influence physiology and reproduction.^7^ Because of long-term adaptation to tropical environmental conditions, the greater ability to withstand high temperatures by zebu cattle compared to taurine cattle is a major distinct feature. Under heat stress challenge, *B taurus* show more pronounced and significant physiological responses (increased body temperature and respiration rates [RRs]) compared to *the B indicus* cattle.^8^ Similarly, adverse effects of heat stress on reproductive functions are more prominent in *B taurus* compared to *B indicus.*^9^, ^10^, ^11^

The differences in the thermotolerance levels between *B indicus* and *B taurus* are attributed to biological mechanisms at cellular, morphological, and behavioral levels. Multiple studies have explored the genetic basis of thermotolerance in cattle. The superior heat tolerance of zebu cattle has been traced to positively selected genes.^12^ Single nucleotide polymorphisms in relevant stress response genes have been associated with the heat tolerance phenotype in cattle.^13^, ^14^, ^15^, ^16^ The specific genotypes present in *B indicus* were also found to contribute toward thermotolerance and reduced impacts of heat stress on reproduction.^9^, ^17^ Genetic variants in the regulatory regions have been found to affect gene expression and consequently influence cellular stress response.^18^, ^19^, ^20^ In addition to genetic changes, epigenetic variations that regulate gene expression patterns are considered potential mechanisms for molecular adaptation to stress in animals.^21^

Epigenetic changes have been recently identified to specifically involve in temperature stress adaptation.^22^ In birds, the thermal stress acclimation was found to be mediated by epigenetically regulated expression of *HSPA1A*.^23^ Expression of *HSP90AA1*, *HSP90AB1*, and *HSPA1A* are regulated by methylation levels in their respective promoter regions during heat stress to Naked Neck chicken.^24^ Similarly, altered DNA methylations are associated with the heat stress response of Holstein dairy cows.^25^ Recently, heat stress-induced changes in the DNA methylation patterns of Nellore (*B indicus*) were compared with Angus (*B taurus*) cattle.^26^ Potential epigenetic mechanisms for skin pigmentation of buffalo adapted to different agro-climatic conditions were recently reported.^27^

In the present study, we explored the potential role of DNA methylation for differences in the thermo-adaptability of Hariana (*B indicus*) and its crossbred, Vrindavani (*B indicus* × *B taurus)* cattle. The *in vivo* heat stress challenge showed that Hariana has better thermotolerance compared to its crossbred, Vrindavani. We generated genome-wide DNA methylation profiles, identified differentially methylated genes (DMG), and compared them with their expression patterns. The results revealed the involvement of DNA methylation in the regulation of heat stress response and long-term adaptation of *B indicus* cattle to higher environmental temperature.

## Results

### Zebu and crossbred cattle show differences in relative thermotolerance

**Fig. 1:**
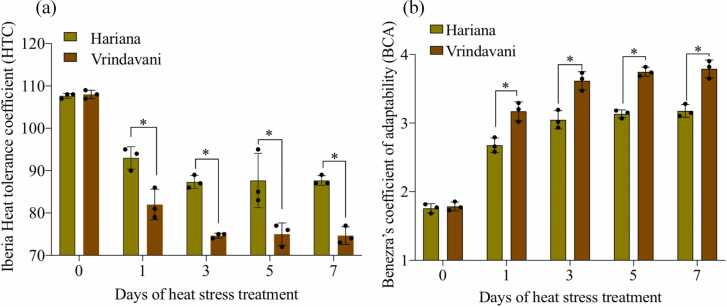
Relative thermotolerance of Hariana and Vrindavani cattle estimated by Iberia heat tolerance coefficient (a) and Benezra's coefficient of adaptability (b) on control day (0 day) and on *in vivo* heat stress treatment days (1, 3, 5, and 7 days). The plotted values are mean and the error bars represent standard deviations (*P < 0.05).

*In vivo* heat stress in a controlled climatic chamber caused stress response in both native and crossbred cattle. The physiological parameters such as rectal temperature (RT) and RR were found to increase on the days of heat stress treatment (1 day, 3 days, 5 days, and 7 days) compared to the day of control (0 day). The highest RT and RR were found on 5 days and 7 days of heat stress treatment. Further, the crossbred cattle (Vrindavani) showed higher values than the zebu (Hariana) cattle, indicating that the crossbred cattle had pronounced heat stress response than the Indian zebu cattle (Table 1). Based on the physiological parameters, the Iberia heat tolerance coefficient (HTC) and Benezra's coefficient of adaptability (BCA) were calculated. Higher HTC values near 100 indicate the animals are more tolerant to heat stress. Accordingly, our results indicate that Hariana cattle had higher HTC and, hence, more tolerant than the crossbred Vrindavani (Figure 1(a)). Further, the lower BCA values indicate better adaptability to heat stress. The BCA values indicate that the Hariana cattle had lower BCA reflecting their better adaptability compared to the Vrindavani cattle (Figure 1(b)).

**Table 1:** Physiological responses (Rectal temperature [RT] and Respiration rate [RR] to heat stress treatment in Hariana and Vrindavani cattle.

	Days/Breed	0 Day	1 Day	3 Days	5 Days	7 Days
RT	Hariana	100.2 ± 0.1	101.7 ± 0.3	102.2 ± 0.1	102.2 ± 0.6	102.2 ± 0.1
	Vrindavani	100.1 ± 0.1	102.8 ± 0.4	103.5 ± 0.1	103.5 ± 0.2	103.5 ± 0.2
RR	Hariana	17.6 ± 1.5	38.3 ± 2.5	46.7 ± 3.1	48.7 ± 1.5	49.7 ± 2.1
	Vrindavani	18.3 ± 1.5	49.3 ± 3.0	59.3 ± 3.1	62.3 ± 1.5	63.3 ± 3.1

### Distinct genome-wide DNA methylation profiles observed in zebu and crossbred cattle

In the whole genome bisulfite sequencing (WGBS), we have generated a total of 238.39 GB of sequence data from 6 samples (3 samples per breed, N = 3) of Hariana and Vrindavani. The analysis of the quality of sequence data indicated that high-quality sequence data were generated and further used for determining the genome-wide methylation status of CpGs at single nucleotide resolution. The mapping of reads to the bovine reference genome (GCF_002263795.1_ARSUCD1.2) indicated an alignment percentage in the range of 60–65 %. The details of data quality and a summary of alignment results are included in Table 2.

**Table 2:** Amount of WGBS raw data and quality, summary of alignment to the reference genome.

Sample name	No of reads	%Q20	%Q30	Clean reads (after quality and adapter trimming)	% Mapped
Hariana 1	276,794,246	99.7	87.66	238,741,492	61.60
Hariana 2	227,893,000	99.73	89.34	205,574,308	60.40
Hariana 3	277,144,968	99.82	91.69	259,019,820	61.70
Vrindavani 1	282,699,792	99.55	84.52	229,333,074	67.80
Vrindavani 2	251,095,404	99.81	92.36	239,054,336	62.00
Vrindavani 3	225,258,778	99.72	89.30	201,964,940	60.02

Abbreviation used: WGBS, whole genome bisulfite sequencing.

Genome-wide DNA methylation patterns of Hariana and Vrindavani samples were obtained and subjected to principal component analysis plot and dendrogram analysis. We found a principal component analysis plot with a correlation matrix showing the grouping of samples as per the cattle breeds (Figure S1(a)). Similarly, the dendrogram analysis revealed the clustering of samples according to the cattle breeds as two separate groups (Figure S1(b)). These results suggested the presence of distinct genome-wide DNA methylation patterns in the Hariana and Vrindavani cattle.

### Differentially methylated CpGs identified between zebu and crossbred cattle

**Fig. 2:**
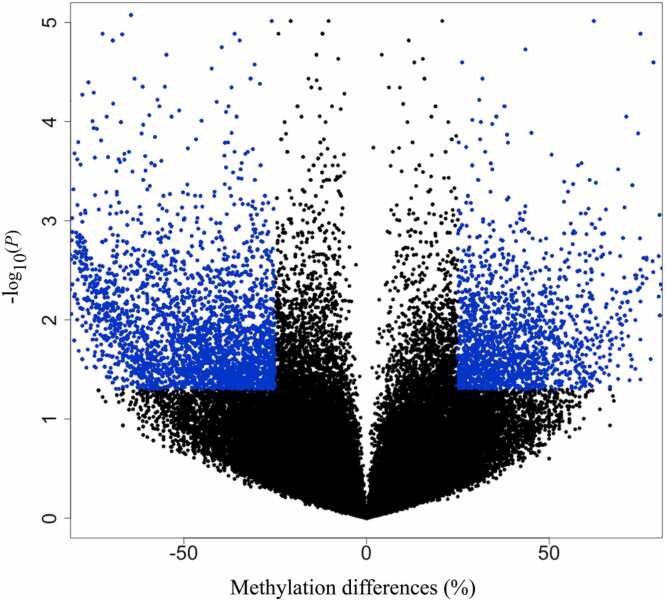
Genome-wide differential methylations estimated between Hariana and Vrindavani cattle estimated after considering methylation difference (25 %) and FDR value (<0.05). Abbreviation used: FDR, false discovery rate.

**Fig. 3:**
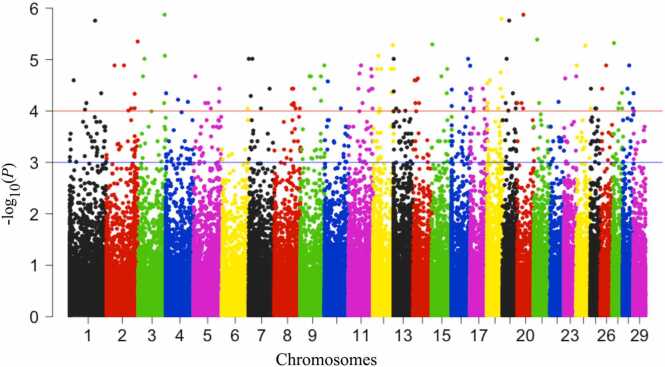
The distribution of differentially methylated CpGs across different chromosomes between Hariana and Vrindavani.

There were a total of 4599 significant differentially methylated CpGs (DMC) identified when a pairwise comparison was made between the groups of Hariana and Vrindavani cattle (with criteria of methylation difference >25 % and false discovery rate [FDR] < 0.05). Among the total DMC, 756 were hypermethylated and 3845 were hypomethylated. The volcano plot shows the identified DMC based on both FDR value and methylation difference (Figure 2). Similarly, the chromosome-wide distributions of CpG sites were shown (Figure 3). Out of these identified 4599 DMC, 3163 were annotated to different genomic regions such as exonic, intronic, promoter/transcription start site (TSS), transcription termination sites (TTS), and intergenic (Figure S2(a)). In addition to single nucleotide resolution information, differentially methylated regions (DMR) were identified by performing a tiling window of 500 bp with a step size of 500 bp. There were a total of 10,723 DMR identified when a pairwise comparison was made between the groups of Hariana and Vrindavani cattle (with criteria of methylation difference >25 % and FDR < 0.05). Among the total DMR, 4575 were hypermethylated and 6148 were hypomethylated. Out of the total DMR (10,723) identified, 10,509 were annotated to different genomic regions such as exonic, intronic, promoter/TSS, TTS, and intergenic (Figure S2(b)). Based on these annotations, top 20 DMG were identified (Table S1).

### DMG involved in different biological process

**Fig. 4:**
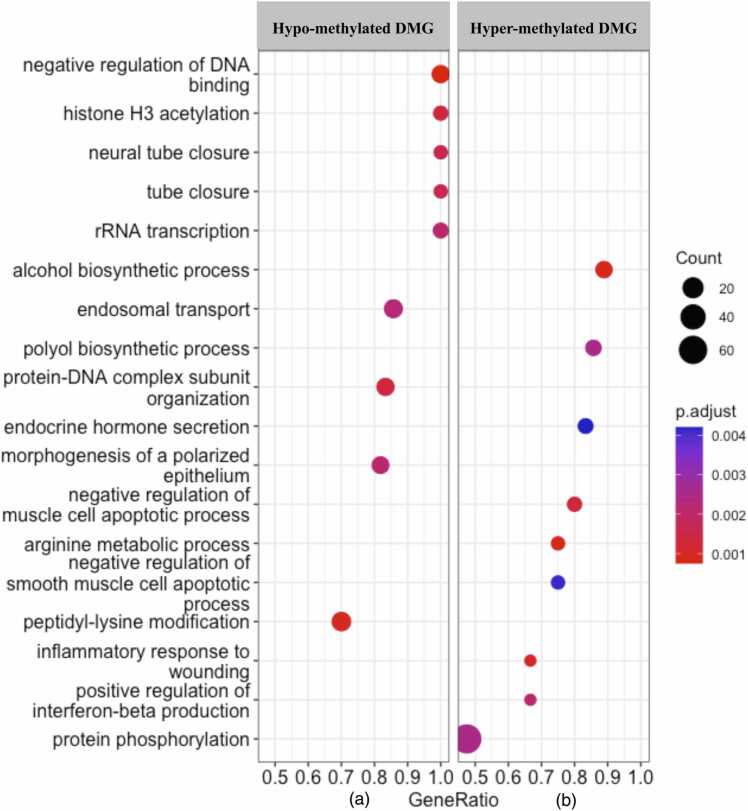
Gene ontology (GO) analysis of differentially methylated genes (DMG). The hypomethylated genes (a) and hypermethylated genes (b) DMG enriched different GO terms.

**Fig. 5:**
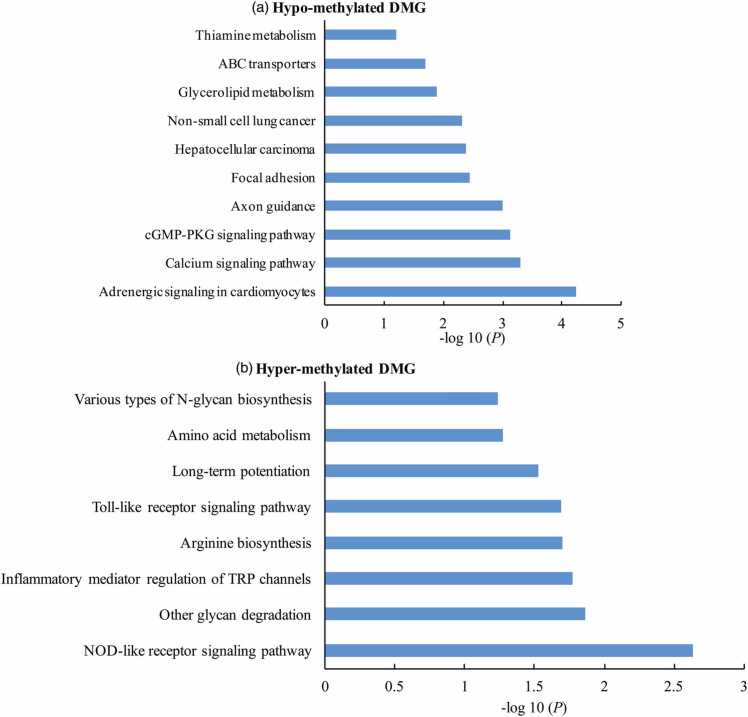
Pathway enrichment analysis of differentially methylated genes (DMG). The hypomethylated genes (a) and hypermethylated genes (b) DMG activated different cellular pathways. Abbreviations used: ABC, ATP-binding cassette; cGMP-PKG, guanosine 3',5'-cyclic monophosphate dependent protein kinase G; TRP, transient receptor potential; NOD, nucleotide-binding and oligomerization domain

The list of hypomethylated and hypermethylated genes was found enriched for different gene ontology (GO) terms. The GO analysis of DMG showed that the biological process was the largest class, followed by the molecular functions. The cellular component is the least enriched and did not come in the top significant GO listed in both hypomethylated and hypermethylated DMGs (Figure 4). Interestingly, in the hypomethylated DMGs, GO terms that are related to epigenetic mechanisms, such as negative regulation of DNA binding, histone H3 acetylation, and protein–DNA complex subunit organization, were found enriched. These biological processes regulate transcription through epigenetic changes. The hypermethylated DMGs enriched some of the important GO terms such as protein phosphorylation, positive regulation of interferon-beta production, smooth muscle apoptotic process, arginine metabolic process, and endocrine hormone secretion. In the kyoto encyclopedia of genes and genomes pathway enrichment analysis, we found 95 pathways at a *P*-value <0.1, out of which 60 were represented by hypomethylated DMG and 35 were represented by hypermethylated DMG. The most enriched top 10 pathways from each of the hypomethylated and hypermethylated DMG were shown in the form of a bar diagram (Figure 5).

### Gene expression and DNA methylation are related to specific genes

Genome-wide expression patterns and comparison between the zebu and crossbred cattle revealed differential expression of genes. There were 1287 differentially expressed genes (DEG), out of which 727 were upregulated and 560 were downregulated in the Hariana cattle compared to the Vrindavani cattle. The comparison between WGBS and RNA-seq analysis revealed the presence of 275 genes that showed both differential methylation and differential expression. Out of these 275 genes, 118 (42.90 %) were found to have a negative correlation between CpG DNA methylation levels and their expression levels. We annotated these genes to different genomic locations and classified them based on the gene expression and DNA methylation patterns (Table 3). The CpG methylation in the promoter or TSS and TTS of specific genes that show different gene expressions were identified and listed in Table 4.

**Table 3:** Annotation of differentially methylated and differentially expressed genes as per their genomic locations.

DNA methylation and expression of genes	Exon	Intron	Promoter	TTS
Hypomethylated and upregulated	5	27	6	3
Hypomethylated and downregulated	13	78	13	6
Hypermethylated and upregulated	5	29	9	4
Hypermethylated and downregulated	7	61	6	3

Abbreviation used: TTS, transcription termination sites.

**Table 4:** List of genes with differential methylation in the promoter region and transcription termination site along with their expression patterns.

DNA methylation and expression	Details of the genes
Hypomethylated in promoter and upregulated expression	*RBM7* (RNA binding motif protein 7)
	*CALM2* (Calmodulin 2)
	*C1H3orf38* (Chromosome 1 C3orf38 homolog)
	*SRP9* (Signal recognition particle 9)
	*TEX12* (Testis expressed 12) *ARHGEF10L* (Rho guanine nucleotide exchange factor 10 like)
Hypomethylated in TTS and upregulated expression	*SF3B5* (Splicing factor 3b subunit 5)
	*OAS1* (2',5'-Oligo A Synthetase 1)
	*RPS21* (Ribosomal protein S21)
Hypermethylated in promoter and downregulated expression	*LOC784451* (C-type lectin domain family 2 member H)
	*EFHD2* (EF-hand domain family member D2)
	*VASP* (Vasodilator stimulated phosphoprotein)
	*CNDP2* (Carnosine dipeptidase 2)
	*NAGA* (Alpha-N-acetylgalactosaminidase)
	*NEDD4L* (NEDD4 Like E3 Ubiquitin Protein Ligase)
Hypermethylated TTS and downregulated expression	*MAPKAPK3* (Mitogen-activated protein kinase-activated protein kinase 3)
	*MMP14* (Matrix metallopeptidase 14)
	*LOC510798* (Tumor necrosis factor receptor superfamily member 26)

Abbreviation used: TTS, transcription termination sites.

### Expression of stress response genes regulated by DNA methylation and microRNA

The differences in the DNA methylations indicate their potential involvement in distinct stress responses, as observed between the Hariana and Vrindavani cattle. We found 79 DMG that belong to the GO term for cellular stress response (GO:0006950). Out of these 79 genes, 46 were hypomethylated, whereas 33 were hypermethylated. Among the identified differentially methylated stress genes, 12 genes also showed distinct expression patterns between Hariana and Vrindavani cattle (Table S2). These genes are important as they may utilize epigenetic regulation for their expression and are also involved in distinct cellular stress responses observed in Hariana and Vrindavani cattle.

Differential methylations within the promoter regions of microRNA genes can regulate their expression levels. We found 12 microRNA genes that had significantly differential methylations in their promoter regions that can regulate their expression. Out of these 12, 9 microRNA had hypomethylation, whereas 3 had hypermethylation. The hypomethylated microRNAs were found to target 74 genes that are differentially expressed. Among these 74 target genes, 52 were downregulated and 22 were upregulated (Table S3). The results show 70.27 % of these target genes expression positively correlated with the methylation patterns in the microRNA genes.

## Discussion

Raising of cattle population with thermotolerance traits is one of the strategies to reduce the impacts of heat stress. In the present study, we compared the *B indicus* and crossbred (*B indicus* × *B taurus*) for heat stress response and involvement of possible epigenetic mechanisms. We found that heat stress caused less perturbed responses (change in RT and RR) in Hariana compared to crossbred Vrindavani cattle. Relative thermotolerance indices (HTC and BCA) extrapolated from these physiological responses indicated that Hariana is more thermo-tolerant than the Vrindavani cattle. Earlier, Beatty *et al*.^8^ found more pronounced responses to heat stress in *B taurus* compared to the *B indicus*. The physiological changes also persisted for a longer time in *B taurus* after the removal of heat stress.^8^ Similarly, Gaughan *et al*.^28^ compared different *B taurus,* crossbred (*B taurus* × *B indicus*) and *B indicus* for thermotolerance traits in the field conditions using panting score associated with heat load index. *B taurus* and crossbred (*B taurus* × *B indicus*) had higher panting score than the *B indicus* cattle.^28^ These observations show that *B indicus* cattle are relatively better adapted to heat stress compared to *B taurus* and crossbred (*B taurus* × *B indicus*).

Genetic variations were associated with differences in the heat stress responses of cattle.^30^, ^29^ These genetic variations have been used for enhancing thermotolerance traits.^31^ However, genetic variations are not entirely responsible for better adaptation to heat stress. Epigenetic modifications are considered as one of the potential mechanisms involved in other production and disease traits of cattle.^32^, ^33^ In this direction, we determined genome-wide DNA methylations in the peripheral blood mononuclear cells (PBMC) of Hariana (*B indicus*) and Vrindavani (*B indicus* × *B taurus*) cattle breeds. The variance partition analysis of genome-wide methylation distribution showed breed-wise separation and clustering, clearly showing that they have distinct genome-wide DNA methylation profiles. Some of these DNA methylation patterns may potentially be associated with differences in the relative thermotolerance earlier observed in Hariana and Vrindavani cattle. Recently, differences in overall methylation patterns were observed for Angus (*B taurus*) and Nellore (*B indicus*) cattle.^26^, ^34^ Similarly, distinct genome-wide DNA methylation patterns observed in different cattle breeds (Holstein cattle, Tibetan cattle and Sanjiang cattle).^35^ These findings collectively indicate that DNA methylation patterns may contribute to differences in some of the phenotypic traits, including adaptation to distinct environmental stressors.

In the comparison between Hariana and Vrindavani, our results show significant numbers of differentially methylations including at single CpG levels (DMC) and at all the CpGs within a region of 500 bp length of genome (DMR). The number of DMC obtained was lesser than the number of DMR obtained. This might be due to the q-value (FDR) cut-off (0.05), which increases because of a large number of CpG subjected to multiple testing corrections. Interestingly, we found relatively higher numbers of hypermethylations in the Vrindavani cattle compared to the Hariana cattle. In the recent study on breed-wise comparison, a higher number of hypermethylations were reported in Angus (*B taurus*) compared to the Nellore (*B indicus*) cattle.^26^ These differences in DNA methylation patterns between *B indicus* and *B taurus* suggest the possible contribution of epigenetic regulations for differences in their phenotypic traits.

In the present study, the integrated analysis of DMGs and DEGs revealed potential candidate genes for stress response and adaptation traits in cattle. Among these, we were particularly interested in four genes: *TOR1B* (torsin family 1member B), *DERL3* (derlin 3), *GCLC* (glutamate–cysteine ligase catalytic subunit), and *PPP1R15A* (protein phosphatase 1 regulatory subunit 15A) as these proteins have a role in cellular stress and related process. *TOR1B*, similar to *TOR1A* codes for a ATPase proteins (Torsin) with molecular chaperone like function to assist in proper folding of proteins.^36^, ^37^ In the immune stimulated B-cells and stress exposed cells, torsin proteins undergo unique proteolytic cleavage to perform chaperone functions in the endoplasmic reticulum.^38^ In the absence of functional torsin proteins, cells experience enhanced protein disruptions under stressful conditions.^39^ These reports, indicate the importance of torsin protein involvement in cellular stress response. However, there are no reports of epigenetic regulations of torsin in cattle.

*DERL3* (Derlin 3) is a functional component of endoplasmic reticulum-associated degradation system for removal of misfolded glycoproteins during stress response.^40^ Epigenetic changes can regulate the expression of *DERL3*. Hypermethylation of promoter CpG islands was shown to reduce the expression of *DERL3.* This effect leads to higher accumulation of one of the *DERL3* target protein *SLC2A1* which intern leads to higher glycolytic metabolism in the affected cells.^41^ Similar hypermethylation-mediated silencing of *DERL3* and its proteosome activities were noticed in long-term alcohol-induced stress in the hepatic tumors.^42^ In the present study, we found hypermethylation of *DERL3* gene and its associated higher expression in Hariana cattle compared to the Vrindavani cattle. These changes may contribute to the effects of heat stress observed in these two cattle breeds.

*GCLC* catalyzes the first step in glutathione synthesis. It is one of the important genes induced by heat shock in addition to oxidative stress response.^43^, ^44^ Supplementation of amino acids during periparturient period induce higher expression of *GCLC* suggesting better antioxidant and immune functions in dairy cattle.^45^ Choline supplementation during heat stress challenge to polymorphonuclear leukocytes of Holstein cows showed higher expression of *GCLC*.^46^ In the present study, we observed differences in the methylation levels of *GCLC* gene and its expression levels between Hariana and Vrindavani cattle, which respond differently to heat stress. There are no previous reports of epigenetic regulation of *GCLC* expression in cattle. However, epigenetic silencing of *GCLC* gene by hypermethylation of its promoter region was observed in human patients of chronic obstructive pulmonary disease.^47^

*PPP1R15A* or *GADD34* is an important protein to overcome stress-induced blockage of protein synthesis and facilitate recovery of cells after stress response.^49^, ^50^, ^48^ *PPP1R15A* was found co-activated by both tumor necrosis factor alpha and heat shock treatment.^51^ In the present study, differences in the expression and methylation levels of *PPP1R15A* between Hariana and Vrindavani cattle were observed. These results indicated the possible role of *PPP1R15A* in adaption to heat stress. In the similar lines, recent report highlighted the significant role of *PPP1R15A* in adaption to acute and chronic stress by serving as molecular memory of integrated stress response.^52^

DNA methylation levels at the genomic locations of microRNA coding sequences can influence their expression. Higher expression and greater sequence conservation of microRNA (miRNA) were observed when the sequences flanking the miRNA coding regions were hypermethylated.^53^ Cellular stress response also involves changes in the expression levels of specific microRNA.^54^ Conserved miRNAs were found to be involved in regulating stress tolerance in plants.^55^ Heat stress was found to influence the expression levels of microRNA in dairy cattle, indicating their functions in the regulation of stress response.^56^, ^57^ In the present study, we found differences in the methylation levels of nine microRNA coding sequences (bta-miR-107, bta-miR-1284, bta-miR-2326, bta-miR-2396, bta-miR-2441, bta-miR-342, bta-miR-411c-5p, bta-miR-6121–3p, bta-miR-885). Further the target genes of these microRNA were also found differentially expressed between the Hariana (*B indicus*) and its crossbred, Vrindavani (*B indicus* × *B taurus*) cattle. Earlier study, reported differences in the correlation patterns between the expression of bta-mir-2898 and the target stress genes (*HSP70*, *HSP60* and *HSPB8)* in Sahiwal (*B indicus*) and its crossbred, Frieswal (*B indicus* × *B taurus*) cattle.^58^ These results show that epigenetic regulations of stress response can occur through changes in the microRNA expression.

Our results of differences in the DNA methylations between native and crossbred cattle indicate that they are induced by specific environmental settings. The stability of these epigenetic changes (intergenerationally and/or transgenerationally stable) and their contribution to adaptive stress response have practical implications for the cattle industry. For example, developmentally stable DNA methylations can be induced by epigenetic programming that ultimately contributes to the phenotype of offspring.^59^, ^60^ Similarly, transgenerationally stable DNA methylation differences can be associated with heat stress tolerance phenotypes and incorporated in the selection programs to build stress-resilient cattle population.^61^, ^62^

## Conclusion

The present study revealed that zebu cattle, Hariana (*B indicus*) is relatively more thermo-tolerant than its crossbred, Vrindavani (*B indicus* × *B taurus*) cattle. These two breeds also show distinct genome-wide methylation patterns that indicate the involvement of epigenetic regulations for observed phenotypic differences including their differences in relative thermotolerance. Gene set enrichment analysis of DMG represented negative regulation of DNA binding, histone H3 acetylation, and protein–DNA complex subunit organization. Integrated analysis of DNA methylation and gene expression revealed differentially methylated and DEG between Hariana and Vrindavani. Some of these key genes are involved in cellular stress response and immune response functions. Overall, the DNA methylation differences identified may potentially contribute to the distinct heat stress responses in Hariana and Vrindavani cattle.

## Materials and methods

### *In vivo* heat stress experiment

Two dairy cattle breeds were included in the experiment: (1) Hariana (Indian native origin, *B indicus*) and (2) crossbred (*B indicus* × *B taurus*), Vrindavani. Three healthy animals for each breed (n = 3) of age 12–18 months’ age, male calves were selected as experimental animals. Experimental animals were subjected to heat stress in a controlled climatic chamber by exposing them to a temperature of 42 °C for 3 h followed by ambient environmental temperature every day consecutively for 7 days.

### Measurement of physiological responses and calculation of coefficients

In the *in vivo* heat stress experiment, RT and RR were recorded at the end of the heat stress treatment. RT was measured using a thermometer placed against the rectum wall for 2 min. RR was recorded visually by counting flank movements and reported as breath per minute. Based on these observations, the Iberia HTC was calculated as HTC = 100 − 10 (RT °F − 101) and BCA was calculated as BCA = RT °C/38.33 + RR/23.

### Sample collection and processing

Blood samples were collected in heparin-coated vacutainers. The PBMC were isolated using Histopaque-1077 based on density gradient centrifugation following manufacturer's standard instructions.

### DNA isolation and library construction

DNA was extracted from PBMC with a DNA extraction kit (NucleoSpin, Takara) according to the manufacturer's protocol, and the concentration of the genomic DNA was determined with a NanoDrop ND-2000 UV spectrophotometer (Thermo Fisher Scientific). The degradation and contamination of genomic DNA was monitored with 1 % agarose gel electrophoresis. Three DNA libraries for each breed were constructed. Genomic DNA (5.0 ug) was spiked with 26 ng lambda DNA and was fragmented to 200 ± 300 bp with DNA fragmentation reagent, followed by end repair and adenylation. Cytosine-methylated barcodes were ligated to fragmented DNA according to the manufacturer's protocol.

The DNA bisulfite conversion was performed using the EZ DNA Methylation Gold kit (Zymo Research, Tustin, CA, USA) according to the manufacturer's instruction. After DNA bisulfite conversion, single-strand DNA fragments were polymerase chain reaction amplified using the KAPA HiFi HotStart Uracil + ReadyMix (2X) (Kapa Biosystems, Wilmington, MA, USA). Library size was quantified by Qubit 12.0 Flurometer (Life Technologies, Carlsbad, CA, USA), and the insert size was checked on an Agilent Bioanalyzer 2100 system (Agilent Technologies, Santa Clara, CA, USA).

### WGBS and data analysis

The libraries from 6 samples were sequenced on an Illumina Hiseq2500 platform, and 150 nt paired-end reads were generated. The bcl2fastq2 conversion software v2.19 was used to convert base call files from a sequencing run into fastq files. The raw fastq files were preprocessed to retain sequence reads with a mean Phred quality (Q-score) >20. The sequence data were further processed to remove 3′ adapter oligonucleotide sequences and trim 10 bases at 5′ end of read1 and read2 using fastp. After filtering the low-quality reads, the clean reads data were aligned to the Ensembl bovine reference genome (*B taurus* UMD_3.1.1), and the bisulfite mapping of methylation sites was performed by Bismark (version 0.12.5). Methylation calls were performed for each cytosine in CpG, CHG, and CHH contexts (where H is A, C, or T).

The differential methylation analysis was done using methylKit (version 1.8.0). At least a coverage of 10× was used for each CpGs site to be considered for further analysis. In addition, the CpG sites that showed no variation across all samples were filtered out. Logistic regression was applied to evaluate the methylation proportion of each CpG between the groups.

Significant DMC sites were identified from each pairwise comparison based on FDR values (FDR < 0.05) and methylation difference of > 25 %. DMC sites were annotated to genomic features using the genomation Bioconductor package (version 1.16.0).

### Genome-wide expression analysis

We obtained expression patterns of genes in the PBMC of Hariana (*B indicus*) and Vrindavani (*B indicus* × *B taurus*) using the RNA sequencing method. Initial steps included quality control, adapter trimming and alignment to the reference genome (ARS-UCD1.2, GCA_002263795.2). The aligned read count files at gene level and transcript levels were obtained using specific annotation files (general transfer format). The differential expression analysis between the breeds was done using DESeq2 protocol. Transcripts per million counts were subjected to shrinkage estimators for dispersions, followed by fold change analysis with a generalized linear model. Benjamini–Hochberg method was applied for the calculation of FDR (Padj) values. The criteria of log2 fold-changes ≥ 1 with padj <0.05 were used for selecting the DEG.

### Association analysis for DMR and DEG

For association analysis, a set of DEG with differential methylation was obtained from the intersection between the set of DMG and the set of DEG. Negative correlations were identified by correlation analysis between the methylation level of DMRs and the expression level of the corresponding genes.

### Functional analysis

Functional network analysis was done to gain biological insights into differentially methylated loci between cattle breeds. Genes annotated from the selected CpG (different methylation level >25 %, significant at FDR <5 %, position <2 kb from TSS) were included in the gene function network analysis and GO enrichment analysis. Ingenuity pathway analysis (Ingenuity Systems, Inc., Redwood City, CA, USA) with its core analysis features was used. Ingenuity pathway analysis categorizes genes based on annotated gene functions and statistical tests for over-representation of functional terms within a gene list using Fisher’s exact Test. The online tool DAVID version 6.8 was used to perform an enrichment analysis of GO-ontology terms.

## Ethics statement

Animal care and experiments were performed as per the standard guidelines. The experiments were approved by the Institutional Animal Ethics Committee (IAEC) and Committee for the purpose of control and supervision of experiments on animals (CPCSEA) under Ministry of Fisheries, Animal Husbandry and Dairying (MoF AH & D) Government of India.

## Author contributions

BS, GS, TD, and BPM designed the work. BS, MTA, and OK performed the experiments. BS, JG, RKG, SKD, and PKG were involved in analyzing the data. BS wrote the manuscript. PC, SKD, and GS edited the final draft. All the authors read and approved the final manuscript.

## Funding and support

This work was supported by the SERB-DST, Govt. of India, CRG Grant no: CRG/2019/005515.

## Declarations of interest

The authors declare the following financial interests/personal relationships which may be considered as potential competing interests: Basavaraj Sajjanar reports financial support was provided by SERB-DST Government of India. If there are other authors, they declare that they have no known competing financial interests or personal relationships that could have appeared to influence the work reported in this paper.

## Data Availability

Data and materials presented in the manuscript will be made available upon request to the corresponding author.

## Appendix A Supplementary material

Supplementary material Fig. S1: Genome-wide DNA methylation profiles of Hariana and Vrindavani cattle classified based on Principal component analysis (PCA) plots (a) and dendrogram-based analysis (b) showing breed-wise distinct grouping of samples.Fig. S2: Annotation of differentially methylated CpGs or DMC (a) and differentially methylated regions or DMR (b) to genomic regions [Intergenic, Introns, Exon, Promoter and transcription termination sites (TTS)].
