# Supplementary figures and images for: Genome-wide DNA methylation profiles regulate distinct heat stress response in zebu (Bos indicus) and crossbred (Bos indicus × Bos taurus) cattle
Source: Cell Stress Chaperones. 2024 Jun 25;29(4):603–14. doi: 10.1016/j.cstres.2024.06.005 (PMC11264184; doi:10.1016/j.cstres.2024.06.005)

**Fig S1**

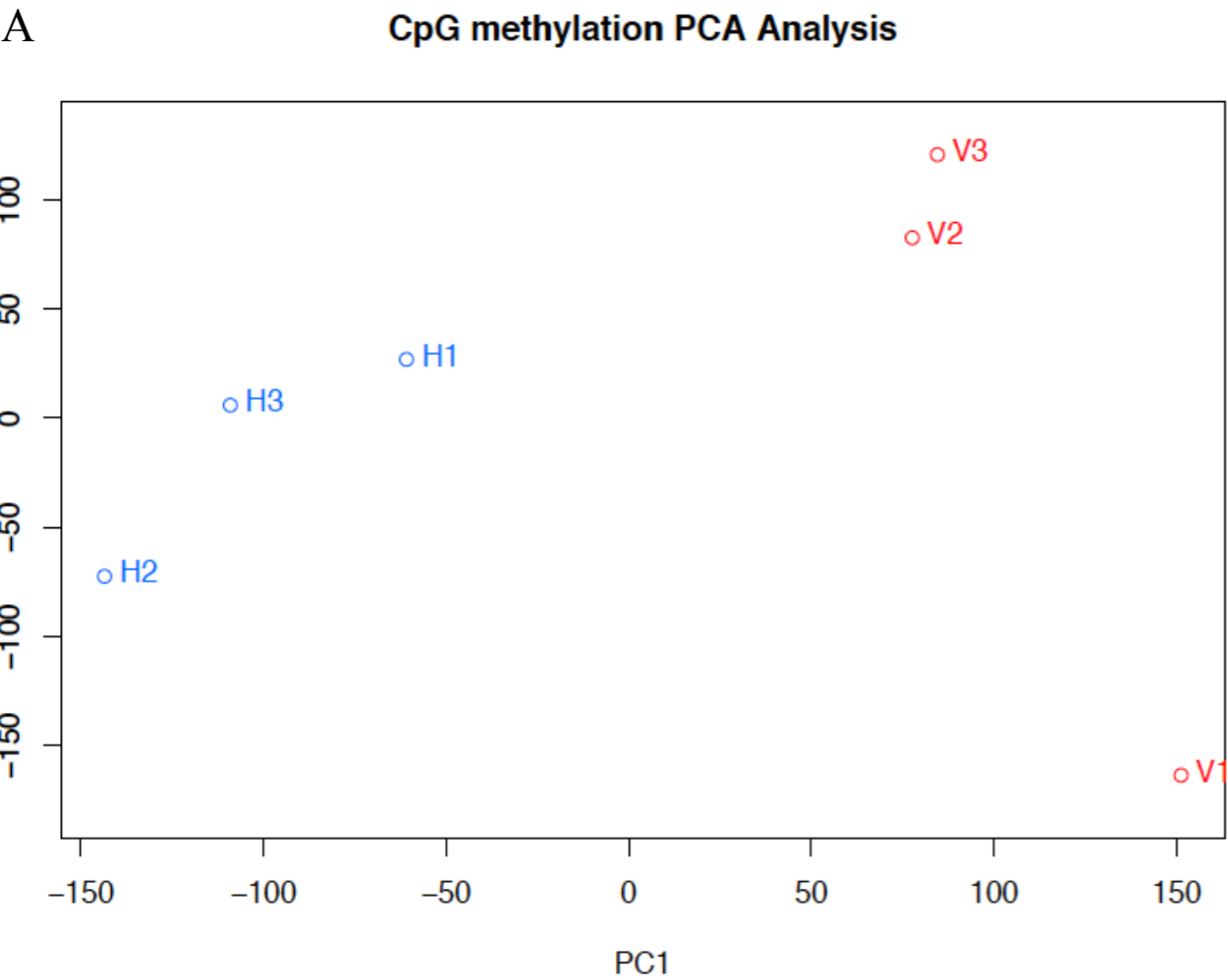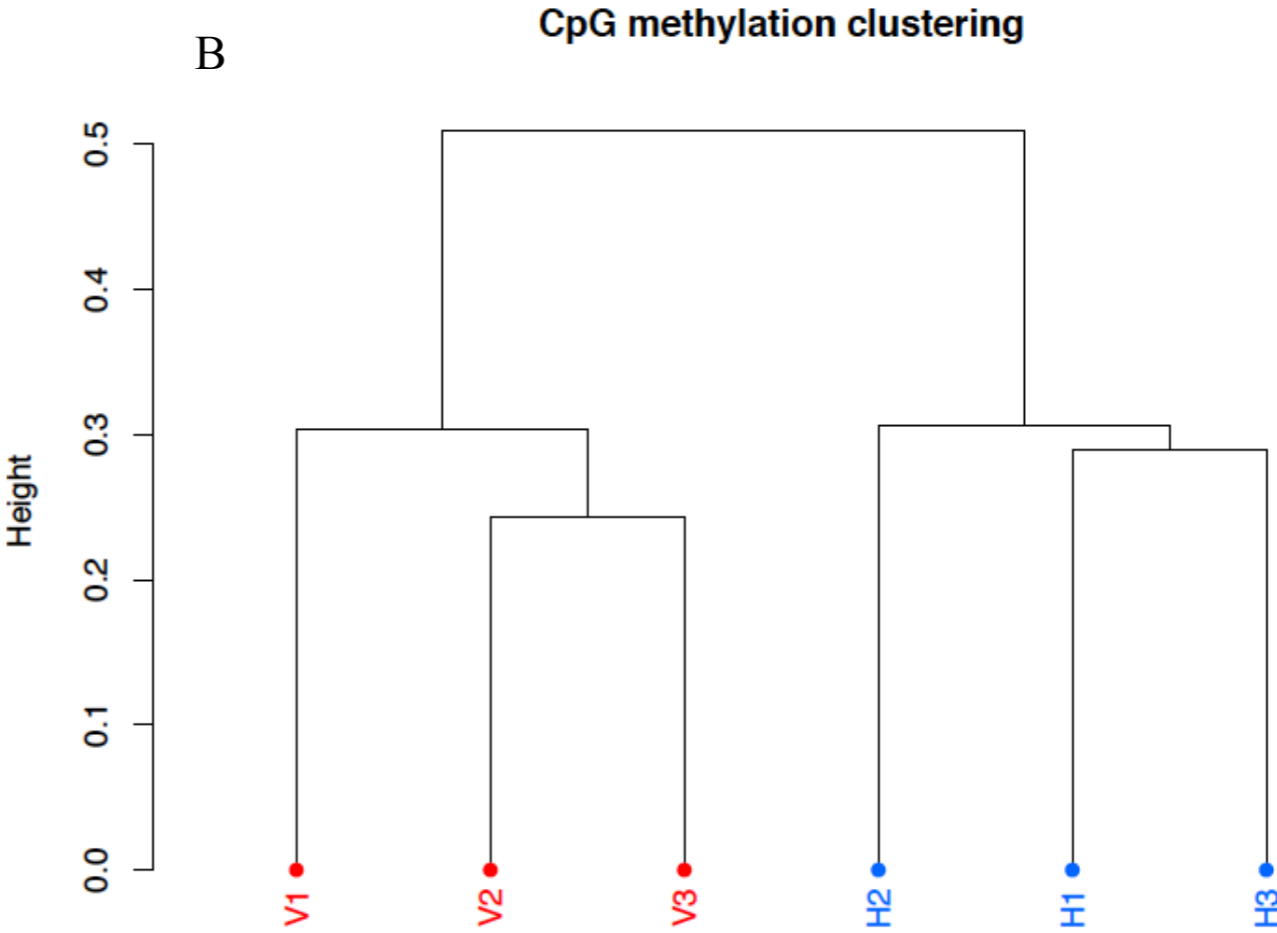

**Fig S2**

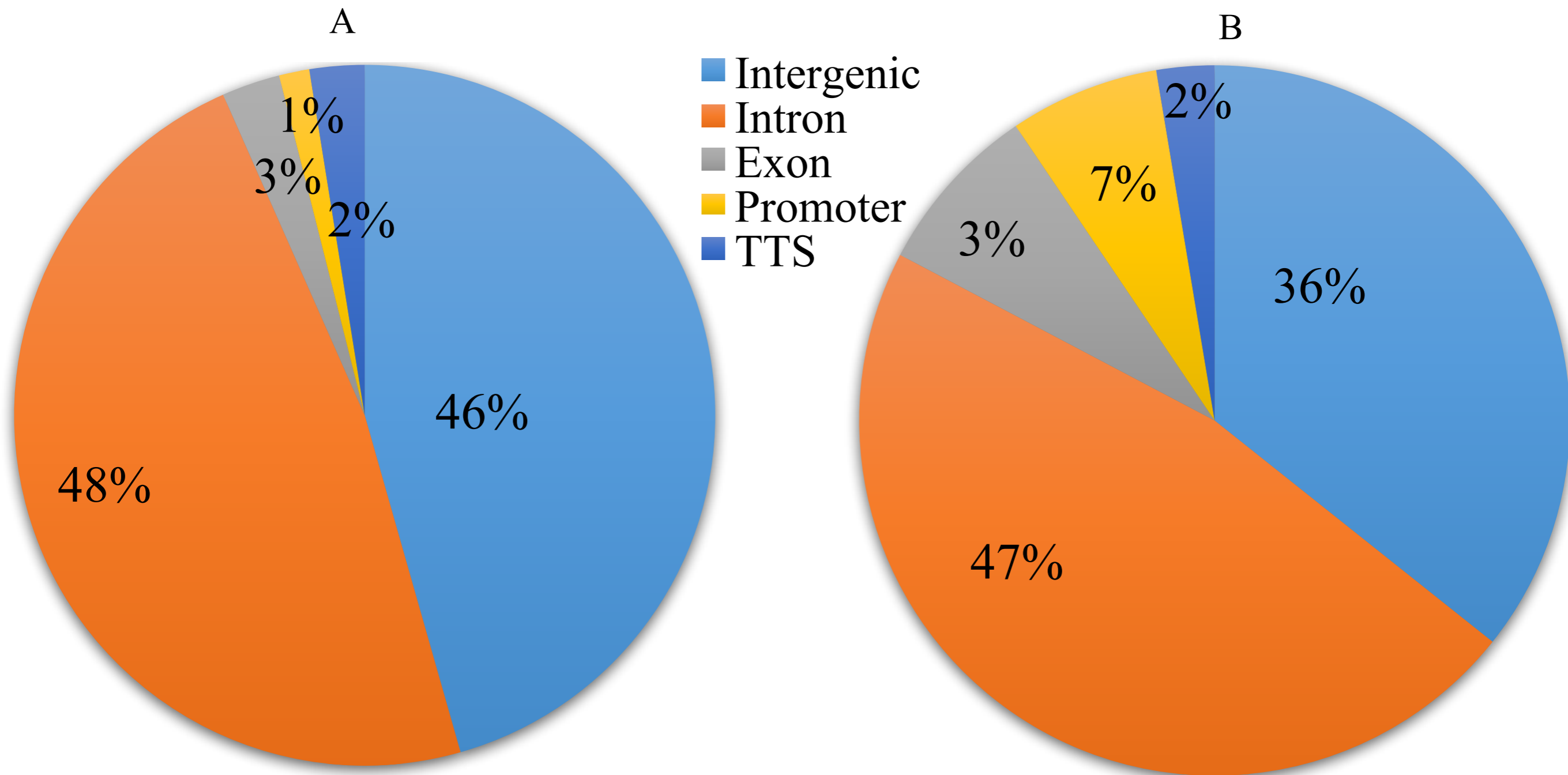

Supplement: Supplementary file 1 — Supplementary material Fig. S1: Genome-wide DNA methylation profiles of Hariana and Vrindavani cattle classified based on Principal component analysis (PCA) plots (a) and dendrogram-based analysis (b) showing breed-wise distinct grouping of samples.Fig. S2: Annotation of differentially methylated CpGs or DMC (a) and differentially methylated regions or DMR (b) to genomic regions [Intergenic, Introns, Exon, Promoter and transcription termination sites (TTS)]. [file mmc1.pdf]
