# Supplementary material for: Genome-wide DNA methylation profiles regulate distinct heat stress response in zebu (Bos indicus) and crossbred (Bos indicus × Bos taurus) cattle
Source: Cell Stress Chaperones. 2024 Jun 25;29(4):603–14. doi: 10.1016/j.cstres.2024.06.005 (PMC11264184; doi:10.1016/j.cstres.2024.06.005)
Supplement: Supplementary file 2 — Supplementary material [file mmc2.docx]

Table S1: List of top 20 genes with significant differentially methylated CpGs between Hariana and Vrindavani cattle

| **Gene name** | **Gene description** | **Methylation (%)** | **q-value** |
| --- | --- | --- | --- |
| *VSIG8* | V-set immunoglobulin domain containing 8 | -80.20 | 4.14E-18 |
| *PRR14L* | Proline rich 14 like | -82.92 | 5.02E-18 |
| *FAM110B* | Family with sequence similarity 110 member b | -78.27 | 3.53E-13 |
| *ZNF704* | Zinc finger protein 704 | -68.46 | 3.10E-11 |
| *C7H19orf57* | Chromosome 7 c19orf57 homolog | -67.89 | 5.74E-11 |
| *RSPH10B* | Radial spoke head 10 homolog B | -59.31 | 1.18E-10 |
| *ZHX2* | Zinc fingers and homeoboxes 2 | -67.08 | 1.45E-10 |
| *EPB41L1* | Erythrocyte membrane protein band 4.1 like 1 | -54.16 | 4.82E-10 |
| *KLK9* | Kallikrein related peptidase 9 | -95.23 | 4.93E-10 |
| *TBC1D2* | TBC1 domain family member 2 | -77.05 | 1.60E-09 |
| *LAMC1* | Laminin subunit gamma 1 | 74.35 | 7.46E-08 |
| *MFN2* | Mitofusin 2 | 69.26 | 5.31E-07 |
| *RABL3* | Rab | 85.18 | 1.11E-06 |
| *PHYHD1* | Phytanoyl-coa dioxygenase domain containing 1 | 65.12 | 1.30E-06 |
| *LOC100851938* | Histone-lysine N-methyltransferase PRDM9-like | 41.07 | 2.90E-06 |
| *LOC112444798* | Small nucleolar RNA SNORA67 | 67.72 | 3.29E-06 |
| *MNS1* | Meiosis specific nuclear structural 1 | 82.97 | 5.62E-06 |
| *MAP2K6* | Mitogen-activated protein kinase kinase 6 | 52.92 | 7.32E-06 |
| *OTUD7A* | OTU deubiquitinase 7A | 93.33 | 9.76E-06 |
| *UPK3B* | Uroplakin 3B | 84.41 | 1.15E-05 |

Table S2: List of cellular stress response genes that show differences in both expression and methylation levels between Hariana and Vrindavani cattle

| **Gene ID** | **Methylation pattern** | **Expression levels**  **(log2FC)** | **Functions** |
| --- | --- | --- | --- |
| *PML* | Hypomethylated | -0.52 | Promyelocytic leukemia gene. It is induced in cell stress response and involved in the control of apoptosis. |
| *GCLC* | Hypomethylated | -1.01 | Glutamate-cysteine ligase catalytic subunit. It is enzyme for glutathione synthesis and involved in oxidative stress response. |
| *PLA2G7* | Hypomethylated | -1.10 | Phospholipase A2 group VII degradation of platelet-activating factor. It is involved in phospholipid catabolism during inflammatory and oxidative stress response. |
| *SMAD7* | Hypomethylated | -1.36 | SMAD family member 7. It acts as antagonist of signalling by TGF-beta and regulate cell growth and differentiation. |
| *CXCL2* | Hypomethylated | -2.90 | Chemokine (C-X-C motif) ligand 2. It is involved in immunoregulatory and inflammatory processes. |
| *PPP1R15A* | Hypermethylated | -2.46 | Protein phosphatase 1 regulatory subunit 15A transcript levels. It is increased following stressful growth arrest conditions. |
| *CCL5* | Hypermethylated | -0.73 | C-C motif chemokine ligand 5. It is involved in immunoregulatory and inflammatory processes. |
| *ADORA2A* | Hypermethylated | -1.47 | Adenosine A2a receptor codes for (G protein)-coupled receptor (GPCR) superfamily. It responds to extracellular cues and activate intracellular signal transduction pathways. |
| *MAP4K4* | Hypermethylated | -1.00 | Mitogen-activated protein kinase kinase 4  serine/threonine protein kinase. It is involved in cellular responses to stimuli and cellular senescence. |
| *TOR1B* | Hypermethylated | 1.32 | Torsin family 1member B. It act as a chaperone and play a role in maintaining the integrity of the nuclear envelope and endoplasmic reticulum. |
| *DERL3* | Hypermethylated | 1.14 | Derlin 3 involved in the degradation of misfolded glycoproteins in the ER. It is up-regulated in response to endoplasmic reticulum stress (UPR). |
| *MAPKAPK3* | Hypermethylated | -1.04 | Mitogen-activated protein kinase-activated protein kinase 3. It is activated by growth inducers and stress stimulation of cells. |

| Table S3: Hypomethylated microRNA and their respective target genes and their expression levels | | | |
| --- | --- | --- | --- |
| **microRNA** | **Target gene** | **Gene description** | **log2FC** |
| bta-miR-107 | TNFAIP3 | TNF alpha induced protein 3 | -1.22 |
|  | SEMA4D | semaphorin 4D | -0.76 |
|  | LGALS9 | galectin 9 | -1.59 |
|  | RBM7 | RNA binding motif protein 7 | 0.68 |
|  | ZFP36 | ZFP36 ring finger protein | -0.91 |
|  | STAT5A | signal transducer and activator of transcription 5A | -0.85 |
|  | ZHX1 | zinc fingers and homeoboxes 1 | 1.12 |
|  | VDR | vitamin D receptor | 1.27 |
|  | FOXJ2 | forkhead box J2 | -0.94 |
| bta-miR-1284 | PPP1R16B | protein phosphatase 1 regulatory subunit 16B | -0.64 |
|  | NDUFS4 | NADH:ubiquinone oxidoreductase subunit S4 | 0.87 |
|  | HCK | HCK proto-onco, Src family tyrosine kinase | -1.42 |
|  | SLC6A9 | solute carrier family 6 member 9 | -1.39 |
|  | CD72 | CD72 molecule | 1.05 |
|  | RABEP1 | rabaptin, RAB GTPase binding effector protein 1 | 0.82 |
|  | STX3 | syntaxin 3 | -1.56 |
|  | APLP2 | amyloid beta like protein 2 | -1.16 |
|  | DHCR7 | 7-dehydrocholesterol reductase | -0.82 |
|  | NEK6 | NIMA related kinase 6 | -1.02 |
|  | RIN2 | Ras and Rab interactor 2 | -1.54 |
|  | HRH2 | histamine receptor H2 | -2.18 |
| bta-miR-2326 | PRAG1 | PEAK1 related kinase-activating pseudokinase 1 | 1.10 |
|  | CSRNP1 | cysteine and serine rich nuclear protein 1 | -0.91 |
|  | DAPP1 | dual adaptor of phosphotyrosine and 3-phosphoinositides 1 | 1.25 |
|  | FKBP1A | FKBP prolyl isomerase 1A | -0.56 |
|  | RELA | RELA proto-onco, NF-kB subunit | -0.90 |
|  | IL27 | interleukin 27 | -1.99 |
|  | MEF2C | myocyte enhancer factor 2C | 1.05 |
|  | IL2RA | interleukin 2 receptor subunit alpha | -1.77 |
|  | TNFRSF1B | TNF receptor superfamily member 1B | -1.61 |
|  | PTP4A2 | protein tyrosine phosphatase 4A2 | 0.72 |
|  | CCL5 | C-C motif chemokine ligand 5 | -0.73 |
| bta-miR-2396 | HPCAL1 | hippocalcin like 1 | -1.88 |
|  | B3GNT5 | UDP-GlcNAc:betaGal beta-1,3-N-acetylglucosaminyltransferase 5 | 1.68 |
|  | FTH1 | ferritin heavy chain 1 | -1.23 |
|  | FGD2 | FYVE, RhoGEF and PH domain containing 2 | 1.36 |
|  | CAMK2D | calcium/calmodulin dependent protein kinase II delta | 0.93 |
|  | VLDLR | very low density lipoprotein receptor | -2.28 |
|  | IL1RN | interleukin 1 receptor antagonist | -2.04 |
|  | VAMP8 | vesicle associated membrane protein 8 | 0.98 |
| bta-miR-2441 | CALM2 | calmodulin 2 | 1.35 |
|  | IRF4 | interferon regulatory factor 4 | -0.88 |
|  | FURIN | furin, paired basic amino acid cleaving enzyme | -0.75 |
|  | CD4 | CD4 molecule | -1.05 |
|  | FST | follistatin | -1.88 |
|  | SYK | spleen associated tyrosine kinase | 1.44 |
|  | IL1RAP | interleukin 1 receptor accessory protein | -2.33 |
|  | MAPKAPK3 | MAPK activated protein kinase 3 | -1.05 |
|  | CCL22 | C-C motif chemokine ligand 22 | -1.34 |
|  | PDXK | pyridoxal kinase | -1.95 |
|  | MGRN1 | mahogunin ring finger 1 | -0.82 |
|  | CYBB | cytochrome b-245 beta chain | 1.10 |
|  | PLPP5 | phospholipid phosphatase 5 | -1.11 |
|  | TEX264 | testis expressed 264, ER-phagy receptor | -1.00 |
| bta-miR-342 | ECM1 | extracellular matrix protein 1 | -1.92 |
|  | MCEMP1 | mast cell expressed membrane protein 1 | -2.19 |
|  | ASRGL1 | asparaginase and isoaspartyl peptidase 1 | -1.97 |
|  | LHFPL2 | LHFPL tetraspan subfamily member 2 | -2.08 |
|  | MTPN | myotrophin | 0.66 |
|  | LARP1 | La ribonucleoprotein 1, translational regulator | -0.64 |
|  | STAT5B | signal transducer and activator of transcription 5B | -0.78 |
|  | PGD | phosphogluconate dehydrogenase | -0.93 |
|  | FAM8A1 | family with sequence similarity 8 member A1 | -1.08 |
|  | CYB561A3 | cytochrome b561 family member A3 | 1.16 |
|  | SLC4A7 | solute carrier family 4 member 7 | 0.71 |
|  | STAT3 | signal transducer and activator of transcription 3 | -0.71 |
|  | IFITM3 | interferon induced transmembrane protein 3 | -1.06 |
| bta-miR-411c-5p | PLAU | plasminogen activator, urokinase | -0.91 |
|  | CTSH | cathepsin H | -0.74 |
|  | FAM111B | FAM111 trypsin like peptidase B | 1.20 |
| bta-miR-6121-3p | SERPINB1 | serpin family B member 1 | -1.18 |
|  | CTSD | cathepsin D | -0.76 |
|  | SATB1 | SATB homeobox 1 | -0.99 |
| bta-miR-885 | ERH | ERH mRNA splicing and mitosis factor | 0.59 |
